# 5-Hydroxymethylation alterations in cell-free DNA reflect molecular distinctions of diffuse large B cell lymphoma at different primary sites

**DOI:** 10.1186/s13148-022-01344-1

**Published:** 2022-10-11

**Authors:** Ye Shen, Jinping Ou, Bo He, Jinmin Yang, Huihui Liu, Lihong Wang, Bingjie Wang, Liang Gao, Chengqi Yi, Jinying Peng, Xinan Cen

**Affiliations:** 1 Department of Hematology, Peking University First Hospital, Beijing, 100034 China; 2 Peking-Tsinghua Center for Life Sciences, Academy for Advanced Interdisciplinary Studies, Peking University, Beijing, 100871 China; 3 State Key Laboratory of Protein and Plant Gene Research, School of Life Sciences, Peking University, Beijing, 100871 China

**Keywords:** Diffuse large B cell lymphoma, Primary sites, 5-hydroxymethylcytosine, Prognosis, Epigenetic biomarkers

## Abstract

**Background:**

5-Hydroxymethylcytosine (5hmC), an important DNA epigenetic modification, plays a vital role in tumorigenesis, progression and prognosis in many cancers. Diffuse large B cell lymphoma (DLBCL) can involve almost any organ, but the prognosis of patients with DLBCL at different primary sites varies greatly. Previous studies have shown that 5hmC displays a tissue-specific atlas, but its role in DLBCLs at different primary sites remains unknown.

**Results:**

We found that primary gastric DLBCL (PG-DLBCL) and lymph node-involved DLBCL (LN-DLBCL) patients had a favorable prognosis, while primary central nervous system DLBCL (PCNS-DLBCL) patients faced the worst prognosis, followed by primary testicular DLBCL (PT-DLBCL) and primary intestinal DLBCL (PI-DLBCL) patients. Thus, we used hmC-CATCH, a bisulfite-free and cost-effective 5hmC detection technology, to first generate the 5hmC profiles from plasma cell-free DNA (cfDNA) of DLBCL patients at these five different primary sites. Specifically, we found robust cancer-associated features that could be used to distinguish healthy individuals from DLBCL patients and distinguish among different primary sites. Through functional enrichment analysis of the differentially 5hmC-enriched genes, almost all DLBCL patients were enriched in tumor-related pathways, and DLBCL patients at different primary sites had unique characteristics. Moreover, 5hmC-based biomarkers can also highly reflect clinical features.

**Conclusions:**

Collectively, we revealed the primary site differential 5hmC regions of DLBCL at different primary sites. This new strategy may help develop minimally invasive and effective methods to diagnose and determine the primary sites of DLBCL.

**Supplementary Information:**

The online version contains supplementary material available at 10.1186/s13148-022-01344-1.

## Introduction

Non-Hodgkin lymphoma (NHL) is a common malignancy of the lymphatic hematopoietic system; it is the seventh most commonly diagnosed malignancy and has the ninth highest mortality rate of all malignancies worldwide, and diffuse large B cell lymphoma (DLBCL) is the most common subtype of NHL [1, 2]. DLBCL behaves highly heterogeneously, and the diversity of its clinical features, cellular morphology, genetic and molecular manifestations indicates that it is an aggregate group of aggressive B cell lymphomas rather than a single clinicopathologic entity [3]. DLBCL can involve almost any organ, but the therapeutic effect and clinical outcome vary according to the primary sites [4–6]. Approximately 30–58% of DLBCL arises in the lymph nodes (LN-DLBCL) and in other lymphoid tissues, such as the thymus, spleen, and Waldeyer’s ring, which are called nodal DLBCL (N-DLBCL). Approximately 25–40% of DLBCLs originate from tissues other than lymphoid tissues; these lymphomas, classified as extranodal DLBCL (EN-DLBCL), include primary gastric DLBCL (PG-DLBCL), primary intestine DLBCL (PI-DLBCL), primary central nervous system DLBCL (PCNS-DLBCL), and primary testicular DLBCL (PT-DLBCL), among others. EN-DLBCL is more aggressive than N-DLBCL [7, 8]. Furthermore, the prognosis of EN-DLBCL patients also differs. It has been reported that PCNS-DLBCL patients had the worst prognosis, with 5-year overall survival (OS) and progression-free survival (PFS) rates of 26.9% and 15.4%, respectively, followed by PT-DLBCL patients, with 5-year OS and PFS rates of 38.2% and 35.3%, while PG-DLBCL patients (5-year OS: 70.3%, 5-year PFS: 64.8%) and N-DLBCL patients (5-year OS: 65.5%, 5-year PFS: 57.0%) had favorable prognoses [4]. The primary site is crucial for determining the clinical features, chemotherapy regimen options and prognosis of DLBCL patients. It is necessary to elucidate the underlying mechanisms in the prognosis of patients with DLBCL at different primary sites to improve the prognosis. To the best of our knowledge, there are few studies on the mechanism of the prognostic difference in DLBCL at different primary sites, and no research has been performed to explore the molecular differences from the epigenetic perspective.

One of the early events that occurs during carcinogenesis is epigenetic alterations, including aberrant DNA methylation and histone modifications [9]. It is well known that the dynamic balance between cytosine methylation and demethylation affects various life processes, such as development and diseases. In mammalian cells, 5-methylcytosine (5mC) can be converted to 5-hydroxymethylcytosine (5hmC) by ten-eleven translocation enzymes [10]. 5hmC is a critical epigenetic marker that regulates gene expression and can affect cell proliferation, differentiation and cancer development [10]. Compared with the inhibitory effect of 5mC in the promoter region, 5hmC is enriched in gene bodies and shows a positive correlation with gene expression [11]. Recent studies charted 5hmC tissue maps by characterizing the genomic distributions of 5hmC in 19 human tissues derived from ten organ systems of European and Chinese ancestry and revealed that 5hmC exhibits tissue specificity, indicating that 5hmC can potentially regulate tissue development and differentiation [12, 13]. Circulating cell-free DNA (cfDNA) is a convenient, fast, minimally invasive and real-time novel biomarker that can reflect the genetic and epigenetic characteristics of tumors [14, 15]. Recent studies have revealed that 5hmC in circulating cfDNA is an important tumor biomarker. For example, 5hmC in circulating cfDNA is not only a biomarker for cancer diagnosis but is also closely related to tumor stage, OS and recurrence in multiple cancer types, such as gastric cancer, lung cancer, hepatocellular carcinoma and esophageal cancer [14–17]. A recent study identified molecular distinctions between DLBCL and follicular lymphoma (FL) by profiling genome-wide 5hmC in circulating cfDNA from newly diagnosed patients with DLBCL and FL [18]. There is also evidence that proves the prognostic value of 5hmC in DLBCL [19]. However, there is no relevant research on effectively determining DLBCL at different primary sites by using noninvasive detection technology.

As a novel epigenetic biomarker, 5hmC plays a crucial role in promoting gene expression and is closely related to tumor stage, OS and recurrence [14–16]. Moreover, 5hmC exhibits tissue specificity and has potential roles in tissue-specific development and various pathological processes [12, 13]. Thus, this study first analyzed whether there were differences in the prognosis of patients with DLBCL at five different primary sites in our center through a retrospective study and then used hmC-CATCH, a previously established bisulfite-free and cost-effective 5hmC detection technology with single-base resolution, to profile the epigenetic alterations underlying molecular differences in the plasma cfDNA of patients with DLBCL at different primary sites to provide new molecular insights [20]. Our results demonstrated that the primary site was associated with clinical characteristics and was a crucial factor affecting the prognosis of DLBCL patients. Moreover, 5hmC in circulating cfDNA can not only reflect the different clinical characteristics of DLBCL but also has significant molecular differences in patients with DLBCL at different primary sites, so it can be used as an effective epigenetic biomarker.

## Results

### Primary sites were associated with the clinical characteristics and outcomes of DLBCL patients

A total of 216 DLBCL patients were included in the retrospective part of this study, including 47 (21.76%) N-DLBCL patients and 169 (78.24%) EN-DLBCL patients; specifically, 47 patients with LN-DLBCL, 56 patients with PG-DLBCL, 57 patients with PI-DLBCL, 28 patients with PT-DLBCL, and 28 patients with PCNS-DLBCL were included. The median ages of patients with N-DLBCL and EN-DLBCL were 60 and 63 years, respectively. Compared with the N-DLBCL group, the EN-DLBCL group had more men than women and presented with higher Eastern Cooperative Oncology Group (ECOG) performance status (PS) scores. Except for sex and ECOG-PS, all clinical features were comparable between N-DLBCL and EN-DLBCL patients, including age, B symptoms, pathological classification and international prognostic index (IPI) score, bulky disease, Ki-67 and lactate dehydrogenase (LDH). The demographic and baseline clinical characteristics of the 216 DLBCL patients are listed in Additional file 1: Table S1.

**Fig. 1:**
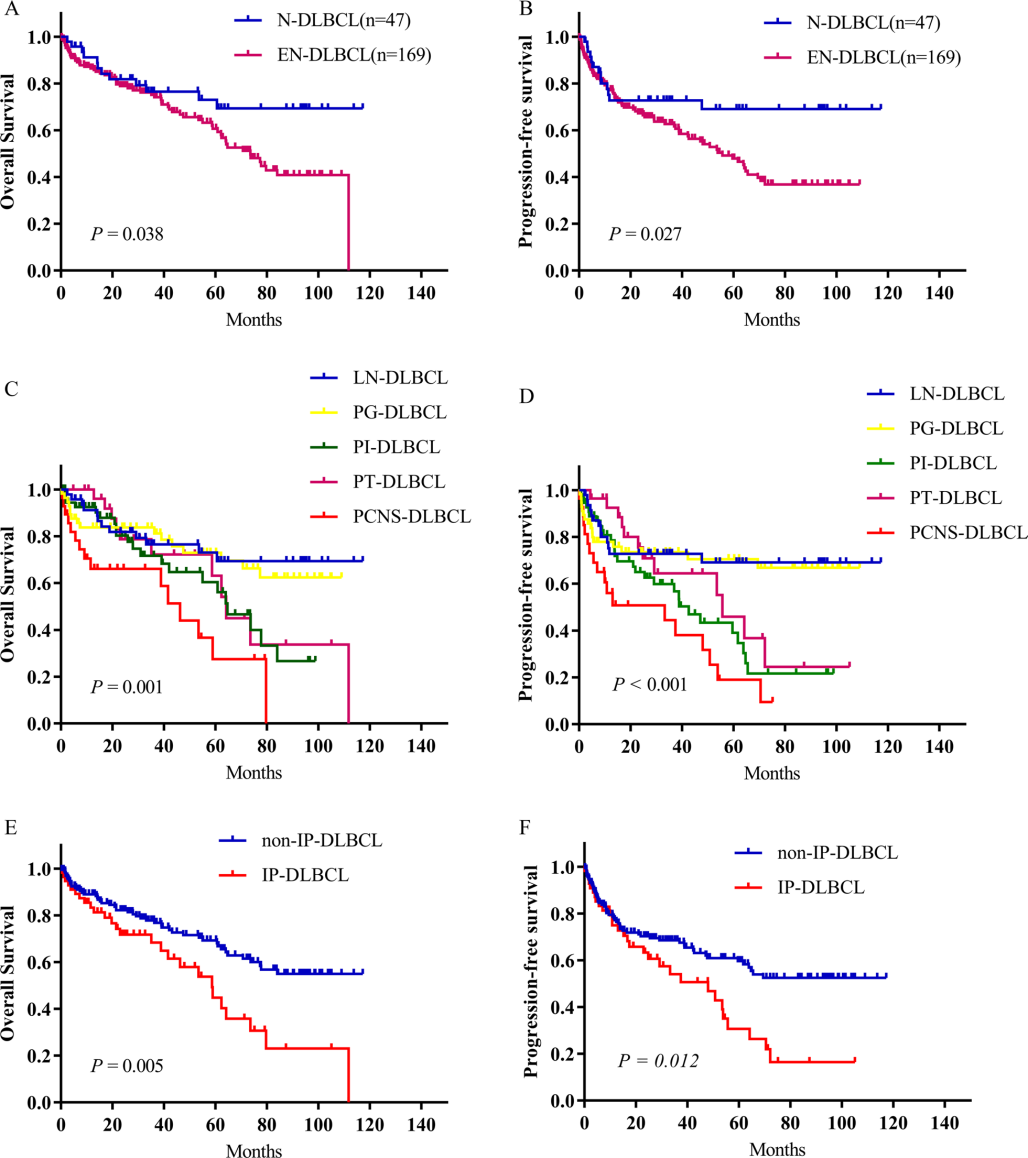
Overall survival (OS) (**A**) and progression-free survival (PFS) (**B**) of patients with nodal diffuse large B cell lymphoma (N-DLBCL) and extranodal DLBCL (EN-DLBCL). OS (**C**) and PFS (**D**) of patients with DLBCL at different primary sites. OS (**E**) and PFS (**F**) of IP-DLBCL patients and non-IP-DLBCL patients. LN-DLBCL, lymph node-involved DLBCL; PG-DLBCL, primary gastric DLBCL; PI-DLBCL, primary intestinal DLBCL; PT-DLBCL, primary testicular DLBCL; PCNS-DLBCL, primary central nervous system DLBCL; and IP-DLBCL: primary immune-privileged site-associated DLBCL

Among these 216 DLBCL patients, the median follow-up time was 56.77 (range 0.33–117.29) months. The 5-year OS and PFS for the entire group were 63.6% and 53.0%, respectively. The 5-year OS rates for N-DLBCL and EN-DLBCL patients were 73% and 60.6%, respectively (*P* = 0.038) (Fig. 1A), and the 5-year PFS rates were 69.1% and 47.9%, respectively (*P* = 0.027) (Fig. 1B). Further analysis was conducted to evaluate the influence of anatomical site on OS and PFS in DLBCL. The results showed significant differences in OS (*P* = 0.001) and PFS (*P* < 0.001) in the different anatomical sites (Fig. 1C and D). LN-DLBCL and PG-DLBCL patients had superior 5-year OS and PFS, whereas PCNS-DLBCL, PI-DLBCL and PT-DLBCL patients had inferior 5-year OS and PFS. According to the traditionally described “immune sanctuary” sites of the central nervous system (CNS) and testes, the 216 DLBCL patients were divided into a primary immune-privileged site-associated DLBCL (IP-DLBCL) group (including PT-DLBCL and PCNS-DLBCL) and a non-IP-DLBCL group (including PG-DLBCL, PI-DLBCL and LN-DLBCL). Patients with IP-DLBCL had a significantly worse prognosis than patients with non-IP-DLBCL (5-year OS rate: 44.8% vs. 69.3%, *P* = 0.005; 5-year PFS rate: 30.7% vs. 59.6%, *P* = 0.012). All data are shown in Table 1.

**Table 1:** OS and PFS of patients with DLBCL in different primary sites

Primary site	n	3-year		5-year	
		PFS (%)	OS (%)	PFS (%)	OS (%)
LN-DLBCL	47	72.7	76.5	69.1	73.0
PG-DLBCL	56	73.5	83.7	70.4	72.9
PI-DLBCL	57	59.8	71.6	39.1	60.3
PT-DLBCL	28	64.4	72.1	46.0	63.1
PCNS-DLBCL	28	44.5	66.1	19.1	27.5
IP-DLBCL	56	54.1	68.3	30.7	44.8
Non-IP-DLBCL	160	68.6	77.8	59.6	69.3

OS: Overall survival; PFS: progression-free survival; DLBCL: diffuse large B cell lymphoma; LN-DLBCL: lymph node-involved DLBCL; PG-DLBCL: primary gastric DLBCL; PI-DLBCL: primary intestinal DLBCL; PT-DLBCL: primary testicular DLBCL; PCNS-DLBCL: primary central nervous system DLBCL; and IP-DLBCL: primary immune-privileged site-associated DLBCL

### The genome-wide 5hmC profiles of cfDNA differed among patients with DLBCL at different primary sites

A total of 20 patients with DLBCL were included in this part of the study, including 5 with LN-DLBCL, 4 with PG-DLBCL, 3 with PI-DLBCL, 5 with PT-DLBCL, 3 with PCNS-DLBCL and 4 healthy individuals as healthy controls (Additional file 1: Table S2). The median age at diagnosis of all the patients was 59 (28–80) years. The age of patients in the LN-DLBCL group, PT-DLBCL group, PCNS-DLBCL group, PI-DLBCL group and PG-DLBCL group was compared with that of the healthy control group, and there was no significant difference (*P* > 0.05). Based on the Hans algorithm [21], 8 (40%) patients were the germinal center B cell-like (GCB) type, and 12 (60%) patients were the non-GCB type. A total of 65.00% (13/20) of DLBCL patients had Ki-67 ≥ 70%. A total of 35.0% (7/20) of patients had elevated LDH levels. According to the IPI score, the 20 DLBCL patients were classified into low-risk (11 patients), low-intermediate-risk (6 patients) and high-intermediate-risk groups (3 patients). For other clinical data, refer to Additional file 1: Table S2.

### Overview of 5hmC from plasma cfDNA among DLBCLs at different primary sites

**Fig. 2:**
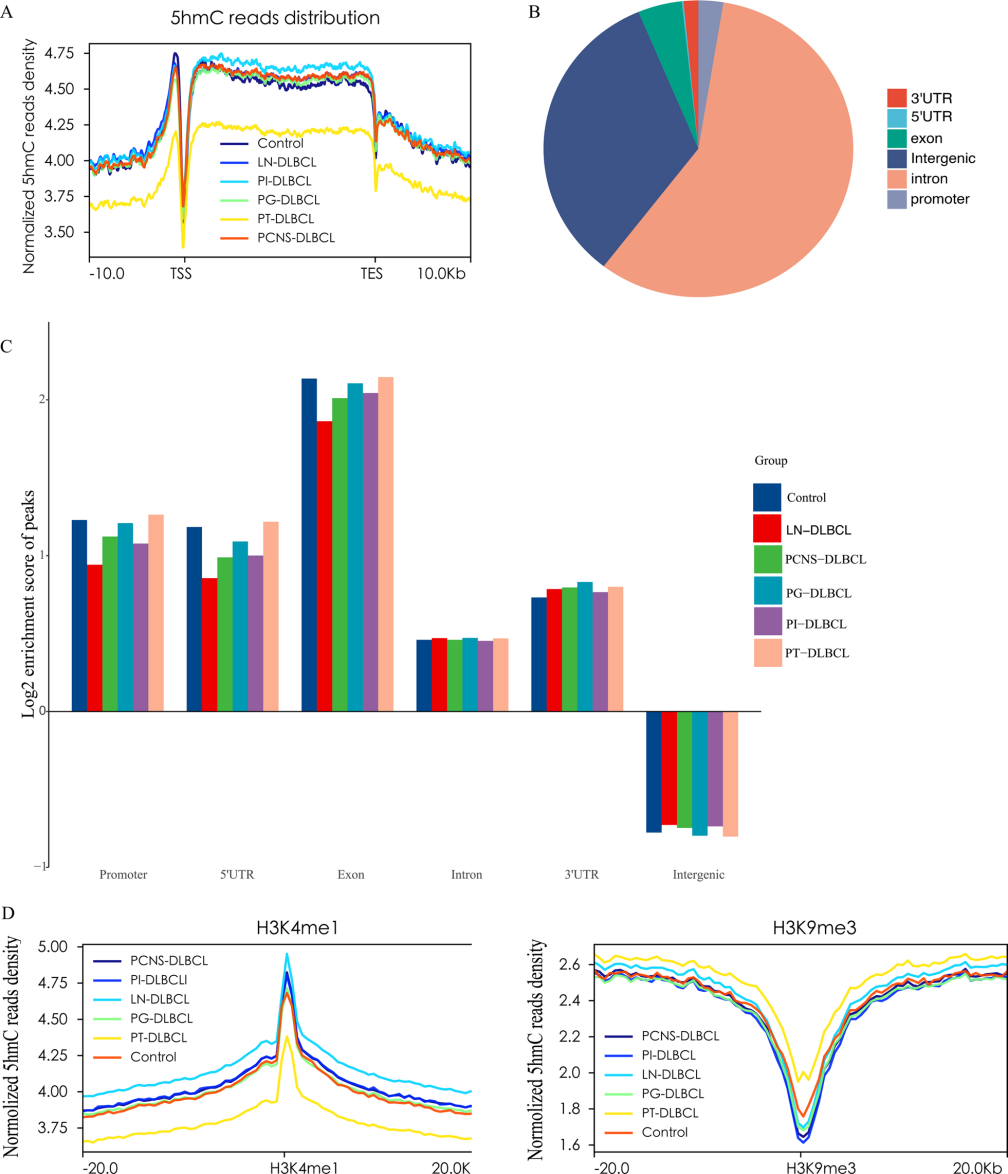
Genome-wide distribution of 5hmC from the plasma cfDNA of individuals in the LN-DLBCL, PT-DLBCL, PCNS-DLBCL, PI-DLBCL, PG-DLBCL and healthy control groups. **A** Metagene profiles of cell-free 5hmC in the six groups. **B** The pie chart shows the overall genomic distribution of hMRs in cfDNA. **C** Normalized enrichment score of hMRs across distinct genomic regions relative to that expected in the 6 groups of samples, with positive values indicating higher than expected enrichment. **D** Profiles of H3K4me1 and H3K9me3 modifications around distal hMRs in the six groups of samples

To explore whether the genome-wide 5hmC profile in circulating cfDNA was distinct in the LN-DLBCL, PT-DLBCL, PCNS-DLBCL, PI-DLBCL, PG-DLBCL and healthy control groups, we profiled the 5hmC signals of the patients and healthy individuals via hmC-CATCH (24 hmC-CATCH samples; average, 39 million paired-end reads, average sequencing depth: ~ 3.7) (Additional file 1: Table S3). We found that the 5hmC-containing spike-in sequence was specifically and efficiently enriched (Additional file 1: Fig. S1A, Additional file 1: Table S4) and displayed a high detection rate for 5hmC (~ 97%) (Additional file 1: Fig. S1B). Furthermore, all the samples displayed a characteristic fragment length distribution with clear nucleosomal periodicity (Additional file 1: Fig. S1C). To improve the confidence of detection, we filtered the reads that did not contain C-to-T conversion signals (Additional file 1: Fig. S1D). This ensured that all peaks were 5hmC-enriched regions. We first compared the distribution of 5hmC along the gene bodies of the six groups and found underrepresentation of 5hmC at the transcription start site (TSS) and transcription termination site (TTS) regions and overrepresentation at gene bodies (Fig. 2A). The PT-DLBCL group displayed the lowest 5hmC level in gene bodies among the six groups. The 5hmC levels were comparable in the remaining 4 DLBCL groups and the healthy control group (Fig. 2A). Furthermore, 5hmC-enriched regions (hMRs) were mainly distributed in introns and intergenic regions (Fig. 2B), and the genome-wide analysis of hMRs showed that hMRs were mostly enriched in exons and promoter regions in gene bodies, whereas hMRs were depleted in intergenic regions (Fig. 2C), which was consistent with the findings of previous studies [22, 23]. In addition, as reported [17, 24], 5hmC tended to be enriched in active histone marks (e.g., H3K4me1, H3K4me3 and H3K27ac), whereas it was depleted in repressive markers (e.g., H3K9me3 and H3K36me3) (Fig. 2D; Additional file 1: Fig. S2A–C). Collectively, these data suggest that 5hmC is preferentially located in gene bodies and active histone modification regions, with potential implications in transcriptional regulation.

### Differential 5hmC genes among DLBCL at different primary sites

**Fig. 3:**
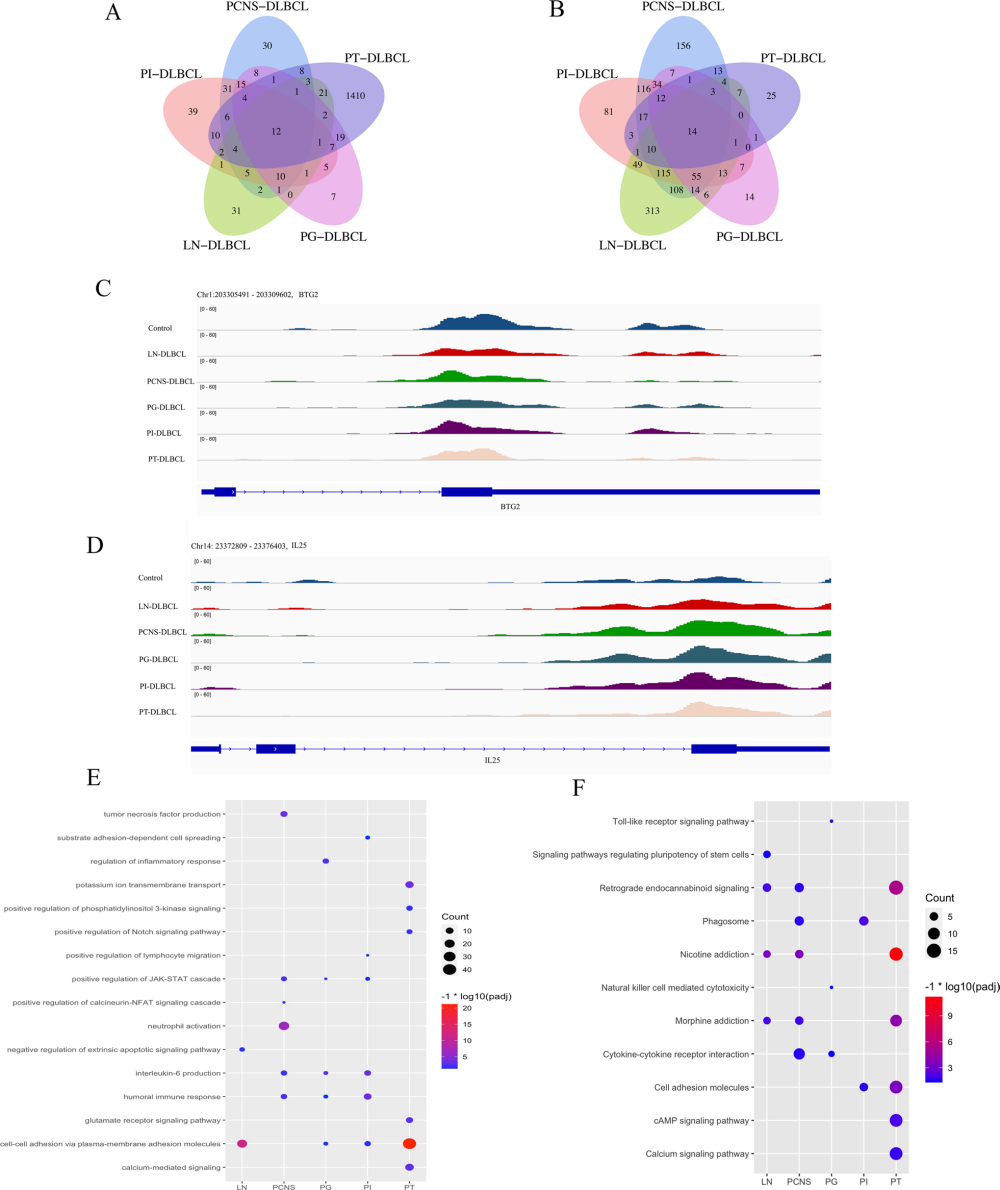
**A** 5hmC-gain genes. **B** 5hmC-loss genes. IGV visualization of the 5hmC signals of the BTG2 gene (**C**) and IL-25 gene (**D**) and surrounding regions. GO biological processes (**E**) and KEGG pathway enrichment analysis (**F**) of the differentially enriched 5hmC genes in DLBCLs at different primary sites

Because 5hmC is positively correlated with gene expression [12], we identified site-specific 5hmC genes in DLBCL at different primary sites (p.adj < 0.05 and |fold change|≥ 1). There were 810, 276, 681, 1623 and 820 differentially enriched 5hmC genes in the LN-DLBCL, PG-DLBCL, PI-DLBCL, PT-DLBCL and PCNS-DLBCL groups, respectively, compared with the healthy control groups. There were 97, 94, 153, 1511 and 141 5hmC-gain genes (Fig. 3A) and 713, 182, 528, 112 and 679 5hmC-loss genes (Fig. 3B). The genes in the PT-DLBCL group mostly showed 5hmC gain, while the genes in the PCNS-DLBCL, PG-DLBCL, PI-DLBCL and LN-DLBCL groups mostly showed 5hmC loss. For example, CXCL13 is a selective B cell chemoattractant, and its function is mediated by the CXCR5 receptor, which is expressed in mature B-lymphocytes, follicular helper T cells and skin-derived migratory dendritic cells. In the context of malignancies, the CXCR5/CXCL13 axis induces proliferation, growth, invasion and migration of malignant cells and correlates with poorer prognosis, more metastasis, and larger tumor size in many cancers [25, 26]. The CXCL13 gene gained 5hmC signal in the PT-DLBCL group in this study (Additional file 1: Fig. S2D). CXCL13 reportedly demonstrates strong cytoplasmic and nuclear expression in primary testicular lymphoma [27]. Furthermore, 42.47%, 7.61%, 17.62%, 88.42% and 22.68% of the differentially enriched 5hmC genes were unique to the LN-DLBCL, PG-DLBCL, PI-DLBCL, PT-DLBCL and PCNS-DLBCL groups. There were 26 differential 5hmC genes that showed 5hmC gain (12 genes) or 5hmC loss (14 genes) in all DLBCLs, regardless of the primary sites (Fig. 3A and B, Additional file 1: Fig. S3A and B). For instance, B cell translocation gene 2 (BTG2) is a p53-induced tumor suppressor gene and has been reported to be involved in tumor proliferation, migration, invasion and other processes [28]; the level of gene body 5hmC signals on BTG2 was decreased in all DLBCL samples compared with healthy control samples (Fig. 3C). In contrast, interleukin 25 (IL-25) is a member of the IL-17 family that can promote and augment T-helper (Th) type 2 responses, and overexpression of IL-25 is involved in lymphoma progression [29]; the level of gene body 5hmC signals on IL-25 was increased in all DLBCL samples compared with healthy control samples (Fig. 3D).

To explore the underlying biological connections of the differentially enriched 5-hydroxymethylated genes, we conducted Gene Ontology (GO) biological process (Fig. 3E) and Kyoto Encyclopedia of Genes and Genomes (KEGG) pathway enrichment analyses (Fig. 3F) in the different DLBCL groups. The results showed that pathways and biological processes enriched in patients with DLBCL at different primary sites not only had the same features but also had their own unique characteristics. For example, “interleukin-6 production” from the GO biological processes was enriched in the PG-DLBCL, PI-DLBCL and PCNS-DLBCL groups. Studies have shown that IL-6, a cytokine induced by NF-κB, promotes DLBCL migration by activating the migration mechanism driven by JAK-STAT, promotes the progression and drug resistance of DLBCL and is related to the poor prognosis of DLBCL patients [30–32]. In addition, the differentially enriched 5hmC genes were also significantly enriched in the “positive regulation of JAK-STAT cascade,” “cell–cell adhesion via plasma-membrane adhesion molecules” and “humoral immune response” in DLBCL of multiple primary sites, indicating that DLBCLs at different primary sites have certain canonical pathways in common. However, in addition to the similarities, PT-DLBCL also had the differential genes enriched in “positive regulation of Notch signaling pathway” and “positive regulation of phosphatidylinositol 3-kinase signaling,” PCNS-DLBCL also had the differential genes enriched in “positive regulation of calcineurin-NFAT signaling cascade,” and PG-DLBCL also had the differential genes enriched in “Toll-like receptor signaling pathway,” suggesting that DLBCLs at different primary sites have their own special pathways. Finally, as reported in other lymphomas [18], enrichment of certain canonical pathways in KEGG, including “retrograde endocannabinoid signaling” and “morphine addiction,” was also identified in this study.

### 5hmC markers from plasma cfDNA can distinguish DLBCL at different primary sites as well as healthy controls

**Fig. 4:**
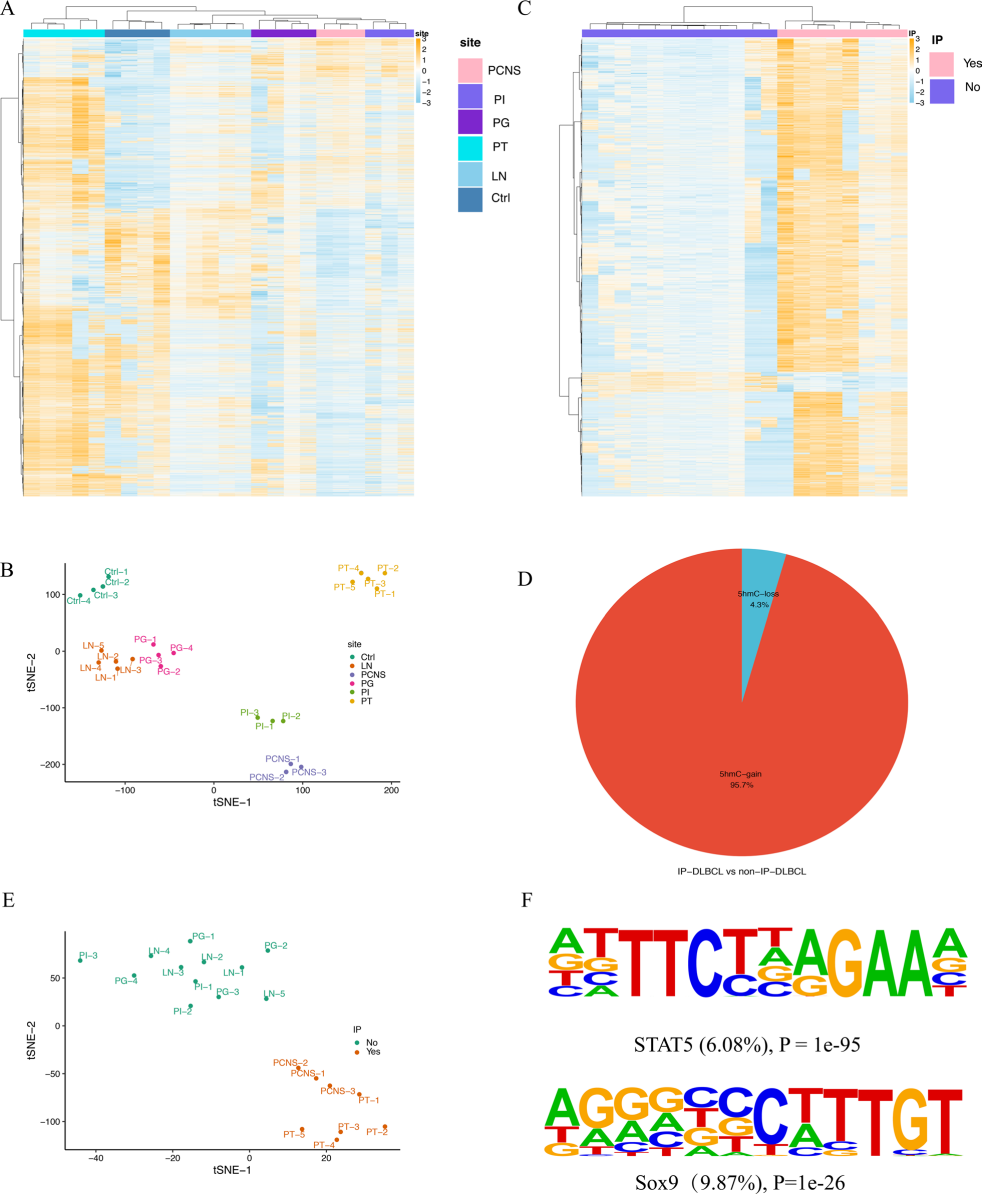
**A** The 40,207 primary sites differential 5hmC regions (psDhMRs) among patients with DLBCL at different primary sites and healthy controls. **B** t-SNE plot of 40,207 psDhMRs in cfDNA among patients with DLBCL at different primary sites and healthy controls. **C** The 38,219 psDhMRs between the IP-DLBCL group and the non-IP-DLBCL group. **D** The pie chart shows 5hmC-gain and 5hmC-loss psDhMRs in the IP-DLBCL group compared with the non-IP-DLBCL group. **E** t-SNE plot of 38,219 psDhMRs in cfDNA between IP-DLBCL and non-IP-DLBCL. **F** The top enriched known transcription factor binding motifs detected in psDhMRs in the IP-DLBCL and non-IP-DLBCL groups. Motif information was obtained from the Homer motif database. The value in parenthesis represents the percentage of target sequences enriched with the binding motif of the indicated transcription factor

To better distinguish DLBCLs at different primary sites, the genome was partitioned into 1 kb bins to identify primary site differential 5hmC regions (psDhMRs) (*P* value < 0.01 and |fold change > 2|). A total of 40,207 psDhMRs among different DLBCL groups were detected (Fig. 4A). For example, the gene body 5hmC signals near metastasis-associated protein 3 (MTA3) on chromosome 2 in the five DLBCL groups showed gain compared with the healthy control group (Additional file 1: Fig. S3C). In these psDhMRs, the PT-DLBCL group mostly showed 5hmC gain, while the PCNS-DLBCL, PG-DLBCL, PI-DLBCL and LN-DLBCL groups mostly showed 5hmC loss and a redistribution of the 5hmC signal (Fig. 4A). In addition, to evaluate the classification effects of 5hmC signals, we carried out t-distributed stochastic neighbor embedding (t-SNE) analysis for the psDhMRs and found that DLBCLs from the same organ system from different donors were clearly clustered together, but DLBCLs from the different organ systems could be readily separated from each other (Fig. 4B). The healthy control samples showed prominent signatures and could be readily separated from the DLBCL samples (Fig. 4B). These results suggest that differential 5hmC markers derived from cfDNA may be effective epigenetic markers for the minimally invasive diagnosis of DLBCL and may help to determine the anatomical site of DLBCL.

### 5hmC markers from plasma cfDNA distinguish IP-DLBCL patients from non-IP-DLBCL patients

Compared with non-IP-DLBCL, IP-DLBCL possesses the biological characteristics of distant recurrence and drug resistance, responds poorly to the current empirical treatment regimens and has inferior outcomes. To explore the difference in 5hmC signaling between IP-DLBCL and non-IP-DLBCL, the 20 DLBCL patients were divided into an IP-DLBCL (PT-DLBCL and PCNS-DLBCL) group and a non-IP-DLBCL (PG-DLBCL, PI-DLBCL and LN-DLBCL) group. Using 5hmC signals can distinguish the IP-DLBCL group and the non-IP-DLBCL group (Fig. 4C), and the 5hmC signals in 36,574 regions were upregulated and those in 1645 regions were downregulated in IP-DLBCL compared to non-IP-DLBCL (Fig. 4D). For instance, the IP-DLBCL group displayed lower gene body 5hmC levels on the phosphatase and tensin homolog (PTEN) gene among the six groups than the non-IP-DLBCL group (Additional file 1: Fig. S3D). Moreover, the t-SNE analysis demonstrated that IP-DLBCL samples could be readily separated from non-IP-DLBCL samples (Fig. 4E).

Furthermore, to investigate the potential mechanism of the difference between the IP-DLBCL group and the non-IP-DLBCL group, motif enrichment analysis was performed (Fig. 4F and Additional file 1: Fig. S4). The results showed that the motif of the transcriptional enhancer factor signal transducer and activator of transcription 5 (STAT5) was significantly enriched in the IP-DLBCL group (*P* = 1e−95) (Fig. 4F); STAT5 is a downstream oncogenic mediator of the JAK-STAT pathway, and the activation of STAT5 promotes tumor progression in various human cancers, especially B cell lymphomas and leukemias [33, 34]. The motif of the transcription factor SOX9 was also significantly enriched in the IP-DLBCL group (*P* = 1e−26) (Fig. 4F); SOX9 is an effective inducer of the formation of stem-like phenotypes, drug resistance, proliferation and invasion and can promote DLBCL development [35, 36]. Therefore, our results indicated that 5hmC potentially interacts with different binding proteins, leading to the difference between IP-DLBCL patients and non-IP-DLBCL patients.

### 5hmC markers from plasma cfDNA can reflect the clinical characteristics of DLBCL patients

**Fig. 5.**
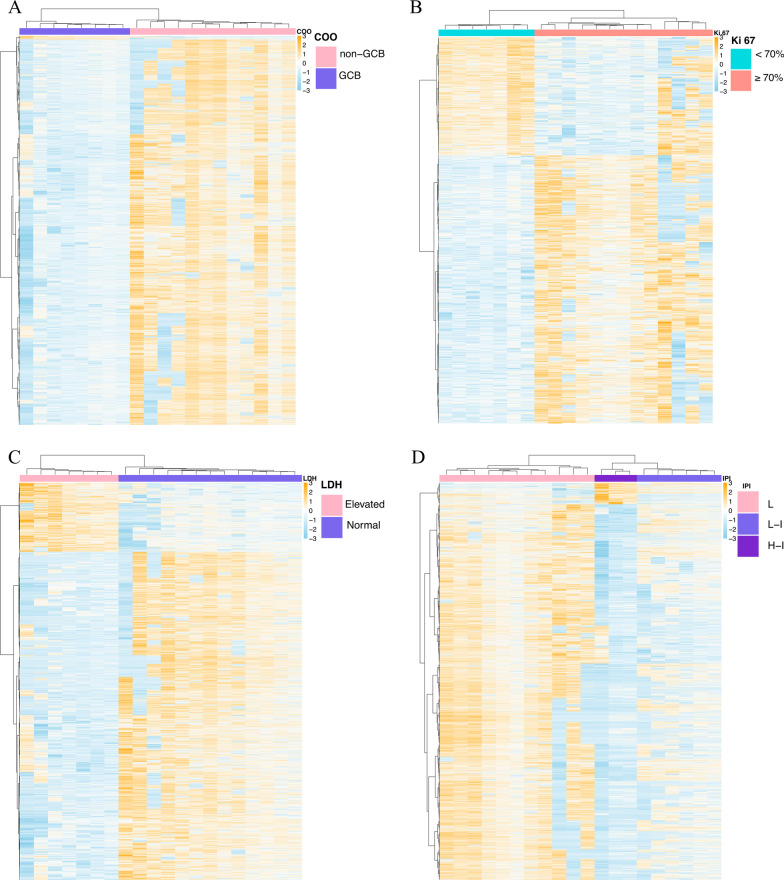
5hmC profiles from cfDNA are shown to reflect the clinical characteristics of DLBCL. **A** Cell of origin (GCB vs. non-GCB, 44,677 DhMRs), **B** Ki-67 (< 70% vs. ≥ 70%, 4876 DhMRs), **C** LDH level (elevated vs. normal, 8815 DhMRs), **D** IPI (low = 0/1 vs. low-intermediate = 2 vs. high-intermediate = 3, 17,239 DhMRs). COO: cell of origin, L: low, L-I: low-intermediate, H-I: high-intermediate

To further explore the potential clinical utility of 5hmC from plasma cfDNA, we further analyzed the correlation between the clinical characteristics of different DLBCL patients and 5hmC signals. The 5hmC profiles in cfDNA not only reflected the patients’ cell of origin but also inferred clinical information about IPI, LDH and Ki-67 (Fig. 5A–C). For example, according to the IPI score, 20 patients with DLBCL were classified into low-risk (11 patients), low-intermediate-risk (6 patients) and high-intermediate-risk groups (3 patients). A total of 17,239 differentially hydroxymethylated regions (DhMRs) were identified among the three groups (Fig. 5D). The 5hmC signals in the high-intermediate-risk group were significantly reduced, followed by the low-intermediate-risk group, and the 5hmC signal in the low-risk group mainly showed 5hmC gain (Fig. 5D), which was consistent with previous reports of increased tumor severity with a lower 5hmC signal [37]. Thus, noninvasive detection of the 5hmC signal in cfDNA can better provide information on the different clinical characteristics of DLBCL and help formulate subsequent treatment plans for DLBCL patients.

## Discussion

DLBCL is highly heterogeneous and aggressive. Except for PCNS-DLBCL patients, rituximab plus cyclophosphamide, doxorubicin, vincristine and prednisone (R-CHOP) is the standard first-line treatment regimen for DLBCL patients. However, the therapeutic effects and clinical outcomes of patients with DLBCL at different primary sites vary greatly. First, this study found that the primary site was an important prognostic factor for DLBCL through retrospective analysis, and LN-DLBCL and PG-DLBCL patients had a favorable 5-year OS and PFS, whereas PCNS-DLBCL patients faced the worst prognosis, followed by PT-DLBCL and PI-DLBCL patients. It has been reported that PCNS-DLBCL patients have an extremely poor prognosis, with a median PFS of 10.2 months, a median OS of 25.3 months, a median OS of 6.1 months after recurrence and a 5-year OS of 38% [38]. The incidence of refractory disease is high, regardless of age. Therefore, it is necessary to reveal the molecular mechanism of poor prognosis and to develop a new generation of induction therapy for PCNS-DLBCL patients. Likewise, PT-DLBCL has a higher tendency to recur, tending to involve the contralateral testis and CNS, with 5-year OS rates of 38.2% to 53.4% [4, 39]. However, PG-DLBCL and LN-DLBCL patients face superior clinical outcomes, with 5-year OS rates of 70.3–73% and 59.8–74.8%, respectively [4, 40, 41]. Interestingly, both PI-DLBCL and PG-DLBCL are located in the gastrointestinal tract, but PI-DLBCL patients have a higher tendency of relapse and refractoriness and have a significantly worse prognosis than PG-DLBCL patients, with 5-year OS rates of 56.9–62.4% [4, 42]. It has been reported that intestinal microecology plays an important role in the occurrence and progression of hematological malignancies, and compared with healthy people, the gut microbiota in patients with DLBCL changes significantly [43]. The possibility that the microenvironmental diversity of different parts of the gastrointestinal tract leads to distinct epigenetic modifications, resulting in prognostic differences, is a concept that merits further exploration. In addition, PCNS-DLBCL and PT-DLBCL patients, with DLBCL arising in the traditionally described “immune sanctuary” sites of the CNS and testes, known as primary immune-privileged site-associated DLBCL (IP-DLBCL), have a significantly worse clinical outcome than non-IP-DLBCL patients according to the anatomical site. IP-DLBCL has previously been shown to share many unique clinical and biological features. In addition to having a relatively poor prognosis and preferential spread to other immune-privileged sites, IP-DLBCL is predominantly of the ABC subtype and shows obvious loss of human leukocyte antigen class I and II expression [44]. Recent studies have found that most IP-DLBCLs harbor CD79B and MYD88 mutations, while these mutations are uncommon in LN-DLBCLs, PG-DLBCLs and PI-DLBCLs, which result in abnormal B cell receptor (BCR) signaling (via CD79B mutation) and oncogenic Toll-like receptor (TLR) signaling (via MYD88 mutation) pathways and confer lymphoma cells an advantage in selective growth at immune-privileged sites [44–47]. In addition, IP-DLBCL showed a high expression rate and copy number increase of 9p24.1/PD-L1, which may be related to tumor immune escape [46]. However, the molecular differences of DLBCLs at different primary sites have not been explained from epigenetic aspects. The present study investigated the epigenetic alterations underlying the molecular differences by mapping the genome-wide profiles of 5hmC in the plasma cfDNA of patients with DLBCLs at different primary sites.

Then, this study revealed the genome-wide 5hmC variation pattern by detecting 5hmC in the plasma cfDNA of patients with DLBCL at five different primary sites. First, we found that DLBCL patients and healthy individuals had prominent differences in 5hmC enrichment in plasma cfDNA. Thus, 5hmC signals may act as biomarkers for the noninvasive detection of DLBCL. Second, 5hmC enrichment among patients with DLBCL at different primary sites differed significantly, and the different sites could be well distinguished by 5hmC signals. This indicates that 5hmC alterations may play a potential role during the formation of DLBCL at different primary sites, and the 5hmC markers derived from plasma cfDNA can serve as effective epigenetic biomarkers not only for the minimally invasive diagnosis of DLBCL but also for determining the primary site. Third, among the identified differential 5hmC genes in the five DLBCL groups compared with the healthy control groups, 42.47%, 7.61%, 17.62%, 88.42% and 22.68% of these genes were unique to the LN-DLBCL, PG-DLBCL, PI-DLBCL, PT-DLBCL and PCNS-DLBCL groups, respectively. This has been similarly reported in esophageal cancer [15]. Fourth, the global loss of 5hmC in PT-DLBCL may be associated with an inferior prognosis. Previous studies proved that the global loss of 5hmC may contribute to tumor progression [14, 37]. Thus, PT-DLBCL and PCNS-DLBCL will show lower 5hmC signals. However, the brain originally had the highest 5hmC signal of any tissue. Hence, we only observed that PT-DLBCL shows the global loss of 5hmC. Moreover, this study found 26 differentially enriched 5hmC genes that showed 5hmC gain or 5hmC loss in all DLBCLs, regardless of the primary sites. Some of these genes (such as the BTG2 gene, MYD88 gene and IL-25 genes) have been found to be closely related to the progression, chemotherapy resistance and inferior OS of lymphoma, and some have become potential therapeutic targets [28, 29, 48]. Finally, the differential 5hmC genes identified in DLBCLs at different primary sites were found to be enriched in several canonical pathways and GO biological processes and had their own unique features as well as similarities. “Interleukin-6 production,” which promotes DLBCL migration by activating the migration mechanism driven by JAK-STAT, was enriched in DCLBCs at multiple primary sites [30–32]. The differential 5hmC genes were also significantly enriched in the “positive regulation of JAK-STAT cascade” in DLBCLs at multiple primary sites, which provides new insights into the activation of the JAK/STAT signaling pathway and indicates that the JAK/STAT signaling pathway may be a potential target of relapsed and refractory DLBCL. Additionally, in the same pathway, we also found that the “positive regulation of phosphatidylinositol 3-kinase signaling” was only enriched in PT-DLBCL, and this has been shown to drive PT-DLBCL cell resistance to ibrutinib [49, 50]. In conclusion, 5hmC markers from plasma cfDNA can readily distinguish DLBCL at different primary sites, which indicated that dysregulation of the 5hmC signal on these tissue-specific DhMRs may be associated with human disease.

IP-DLBCL patients have been proven to have many unique clinical and biological features. If the molecular alterations underlying the poor prognosis of IP-DLBCL can be explained from an epigenetic perspective, new ideas and targets for prevention and treatment may be provided to improve the prognosis of IP-DLBCL patients. In this study, we found that IP-DLBCL patients and non-IP-DLBCL patients had prominent differences in 5hmC enrichment from plasma cfDNA, and IP-DLBCL could be well separated from non-IP-DLBCL patients by the 5hmC signal, indicating that the 5hmC marker originating from plasma cfDNA is a biomarker for the minimally invasive diagnosis of IP-DLBCL. Additionally, the potential interacting binding proteins targeted to differentially modified 5hmC regions may play an important role in IP-DLBCL. These proteins have been reported to be closely associated with tumorigenesis, progression, chemotherapy resistance and inferior prognosis in multiple cancers, including DLBCL [33–36, 51, 52]. Therefore, our results indicated that IP-DLBCL patients can be readily distinguished from non-IP-DLBCL patients by 5hmC signals. IP-DLBCL patients and non-IP-DLBCL patients showed apparent differences in both 5hmC signals and their potentially interacting binding proteins. This study will provide new molecular insights into IP-DLBCL from an epigenetic perspective.

Nevertheless, our study still has limitations. The results of this study showed that the primary site is an important factor affecting the prognosis of DLBCL patients, and there were significant differences in 5hmC from the plasma cfDNA in patients with DLBCL at different primary sites. However, due to the short follow-up time and few outcome events, the relationship between the difference in 5hmC and prognosis was not analyzed in patients with DLBCL at different primary sites. In the future, more plasma and tissue samples need to be included to analyze the 5hmC alterations in patients with DLBCL at different primary sites and explore their potential biological significance.

In conclusion, our results suggested that primary sites are an important factor affecting the prognosis of patients with DLBCL, and the 5hmC markers derived from cfDNA can serve as effective epigenetic biomarkers not only for the minimally invasive diagnosis of DLBCL but also for determining the primary site. This new strategy may help develop minimally invasive and effective methods to diagnose and determine the primary sites of DLBCL.

## Materials and methods

### Participants and study design

The first part of the study retrospectively included 216 DLBCL patients diagnosed and treated at Peking University First Hospital from January 2010 to December 2020, including 47 patients with only lymph node involvement, 56 patients with primary gastric involvement, 57 patients with primary intestinal involvement, 28 patients with primary testicular involvement and 28 patients with primary central nervous system involvement. The collected clinical characteristics and pathological data included age, gender, LDH, ECOG-PS, IPI, GCB and non-GCB subtypes according to the Hans algorithm [21], Ki-67, etc. Most patients were treated with rituximab, cyclophosphamide, doxorubicin, vincristine and prednisone (R-CHOP) and received 18F‐fluorodeoxyglucose positron emission tomography/computed tomography (18F‐FDG PET/CT) monitor and assess treatment response of lymphomas. The primary endpoints of this study were OS and PFS. OS was calculated from the time of diagnosis until the time of death from any cause or until the last follow-up time point. PFS was defined as the time from diagnosis to the time of disease progression, recurrence, death due to any cause, or until the last follow-up time point.

The purpose of the second part was to map the 5hmC genome in circulating cfDNA through next-generation sequencing to explore whether there are molecular differences from 5hmC alterations in patients with DLBCL at different primary sites and whether 5hmC can be used as a biomarker. For inclusion, participants were over 18 years old, met the World Health Organization’s classification of lymphoid neoplasms, did not receive any lymphoma treatment before enrollment [53] and had imaging evidence of involvement of only the lymph nodes (LN-DLBCL), stomach (PG-DLBCL), intestine (PI-DLBCL), testis (PT-DLBCL) or central nervous system (PCNS-DLBCL). Patients were excluded if they were concurrently diagnosed with other tumors. Ultimately, 20 patients, including 5 LN-DLBCL patients, 4 PG-DLBCL patients, 3 PI-DLBCL patients, 5 PT-DLBCL patients, 3 PCNS-DLBCL patients and 4 healthy individuals as healthy controls, were recruited for the study in the Hematology Department of Peking University First Hospital from December 2018 to July 2020. All healthy individuals had no malignant disease history. Pretreatment evaluations included the demographic characteristics and clinical features, such as age, gender, LDH, ECOG, IPI and Ki-67. The study was conducted in accordance with the Declaration of Helsinki and was reviewed by the institutional ethics review committee of Peking University First Hospital.

### Peripheral blood collection and preparation of cfDNA

Peripheral blood from patients and healthy controls was collected for cfDNA preparation. Nine milliliters of blood were collected into EDTA-coated vacuum blood collection tubes. Plasma was prepared by centrifuging twice at 1350×*g* for 12 min at 4 °C and at 13500×*g* for 12 min at 4 °C within 4 h of sample collection. CfDNA was extracted using the Circulating Nucleic Acid Kit (Qiagen, Cat. No. 55114). The fragment size of all the cfDNA samples was verified by the Agilent 4150 TapeStation system. Then, the qualified samples were stored at − 80 °C.

### 5hmC library construction and high-throughput sequencing

The 5hmC libraries of all samples were constructed using high-efficiency hmC-CATCH technology [20]. Briefly, endogenous 5-formylcytosine (5fC) was blocked by EtONH2. The 5hmC was then oxidized to 5fC with K2RuO4. For 5fC newly generated by the oxidation of 5hmC, we achieved selective labeling using the azide derivative of 1,3-indoloquinone. Because adducts were read as T rather than C during PCR, this C-to-T conversion was used as a direct readout of 5hmC. In addition, the azide group allows biotin binding and pull-down, thereby enriching 5hmC-containing DNA for detection. Finally, sequencing was performed on the Illumina HiSeq X Ten platform.

### Mapping and differentially modified region detection

All raw sequencing data were trimmed using Trimmomatic (version 0.32) [54]. Adapter sequences and low-quality sequences at the end of the sequences were trimmed off, and the remaining paired-end reads were mapped to the human genome (version hg19) using Bismark (version 0.15.0) [55] and then filtered with Picard (version 1.119, http://broadinstitute.github.io/picard) to remove polymerase chain reaction (PCR) repeats. hMRs were identified using MACS (version 2.1.1) [56]. The hMRs present in at least two biological replicates were considered high-confidence hMRs.

We then merged all hMRs from the 24 samples to obtain the total hMRs using “Bedtools merge.” Then, the read counts in each merged hMR of all samples were calculated by “Bedtools multicov” and normalized by RPKM. We merged all biological replicates from the same primary sites to enhance the signals. We used the Poisson distribution to estimate the p value of each peak in a tissue. The probability of read counts in each peak of one tissue was estimated by the one-tail Poisson distribution with the parameter as the mean for other primary sites. The p values were further adjusted by the Bonferroni method. The peaks with adjusted p values less than 0.05 and fold changes greater than 2 were regarded as significant primary site-specific differential 5hmC regions (psDhMRs).

FindMotifsGenome.pl of HOMER (version 4.11) was performed to find the corresponding binding proteins targeted to the DhMRs between the primary immune-privileged site-associated DLBCL (IP-DLBCL) and non-IP-DLBCL groups. For the result of motif enrichment in DhMRs, according to the enriched *P* value and the percentage of target sequences enriched with the binding motif, which indicated transcription factors, the top enriched known transcription factor binding motifs were identified, following the approach of Zhang et al. [23].

### Detection of differentially expressed genes and functional enrichment analysis

Genomic annotations of the high-confidence hMRs were performed using BEDtools (version 2.29.2) [57]. The differentially enriched 5-hydroxymethylated genes were identified using the DEseq2 (version 1.20.0) package [58] in R (version 3.6.0) with the criteria |log2foldchange > 1|and P.adj < 0.05. Hierarchical clustering and heatmap analysis were performed by Pheatmap (version 1.8.0) in the R package. Dimensionality reduction by t-SNE in the R package was performed for the analysis and visualization of DhMRs. All paired-end reads were converted into the bedgraph format normalized by bam2bedgraph (version 1.0.4) [57]. The 5hmC site signals on the differentially enriched 5-hydroxymethylated genes were visualized using Integrated Genomics Viewer (IGV) (version 2.5.3) [59]. To explore the underlying biological connections of the differentially enriched 5-hydroxymethylated genes, we conducted Kyoto Encyclopedia of Genes and Genomes (KEGG) pathway enrichment analysis and Gene Ontology (GO) biological processes analysis using the clusterProfiler R package (version 3.12.0) in R package. Subsequently, the top 6 KEGG pathways or top 6 GO biological process terms possibly associated with tumors were selected for display.

### Integration with histone modification data

Chromatin state data based on four histone modifications (H3K4me1, H3K27ac, H3K4me3, H3K9me3 and H3K36me3) were downloaded from the Encyclopedia of DNA Elements (ENCODE) project [60]. The overlap between 5hmC peaks and different chromatin states was calculated by BEDtools (version 2.29.2) [57].

### Statistical analysis

In addition to the bioinformatics analysis described above, the following methods were used in the first part of the study. Analyses of the frequencies were performed using the Pearson *χ*^2^ test for 2 × 2 tables. The OS and PFS of the subgroups were estimated using the Kaplan–Meier method and compared by the log-rank test. The statistical analyses were conducted by IBM SPSS Statistics 22.0 software or GraphPad Prism 9. Differences were considered statistically significant at *P* < 0.05.

## Supplementary Information

**Additional file 1:** **Figures S1–S4. Tables S1–S4.** **Table S1.** Demographic and baseline clinical characteristics of patients with N-DLBCL and EN-DLBCL. **Table S2.** Clinical characteristics of DLBCL patients and healthy controls. **Table S3.** Sequencing technical details including sequencing depth, mapping rate, and PCR duplication rates in 24 cases. **Table S4.** The details of the probe sequences.

## Data Availability

The raw sequence data reported in this paper have been deposited in the Genome Sequence Archive for Human, as a part of GSA in the National Genomics Data Center, under accession number HRA003025 that is publicly accessible at https://ngdc.cncb.ac.cn/gsa-human/. All other data supporting the findings of this study are available from the corresponding author on reasonable request.
