# Supplementary material for: 5-Hydroxymethylation alterations in cell-free DNA reflect molecular distinctions of diffuse large B cell lymphoma at different primary sites
Source: Clin Epigenetics. 2022 Oct 11;14:126. doi: 10.1186/s13148-022-01344-1 (PMC9555108; doi:10.1186/s13148-022-01344-1)
Supplement: Supplementary file 1 — Additional file 1: Figures S1–S4. Tables S1–S4. Table S1. Demographic and baseline clinical characteristics of patients with N-DLBCL and EN-DLBCL. Table S2. Clinical characteristics of DLBCL patients and healthy controls. Table S3. Sequencing technical details including sequencing depth, mapping rate, and PCR duplication rates in 24 cases. Table S4. The details of the probe sequences. [file 13148_2022_1344_MOESM1_ESM.docx]

Supplementary information


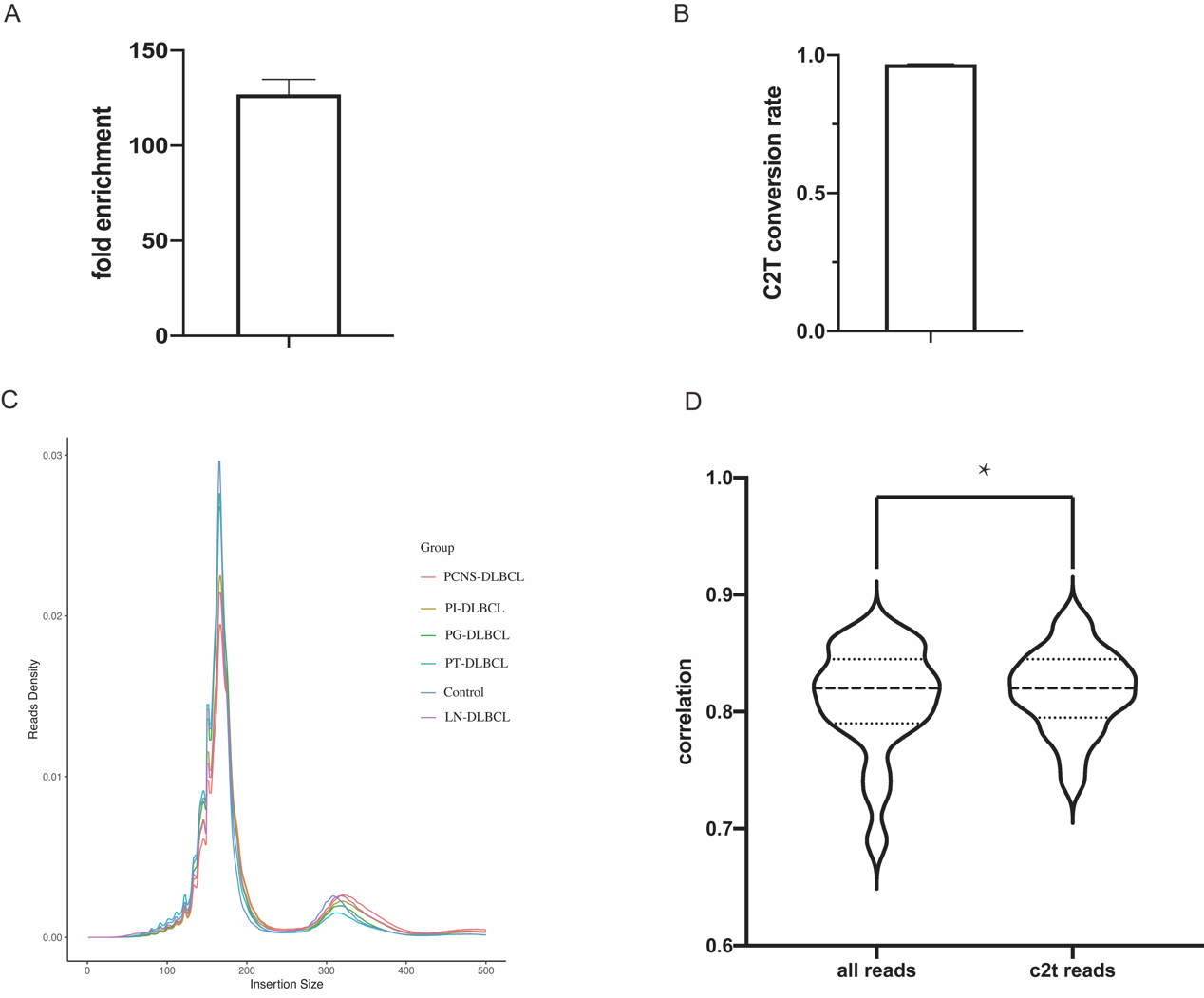


Figure S1. A. Enrichment of spike-in probes. B. The oxidation efficiency in 5hmC and 5fC spike-in probes by high-throughput sequencing. C. Distribution of fragment sizes for each primary site from aligned, filtered and deduplicated reads. D. Pearson’s correlation of biological replicates using total reads or filtered reads (* represents P value< 0.05)


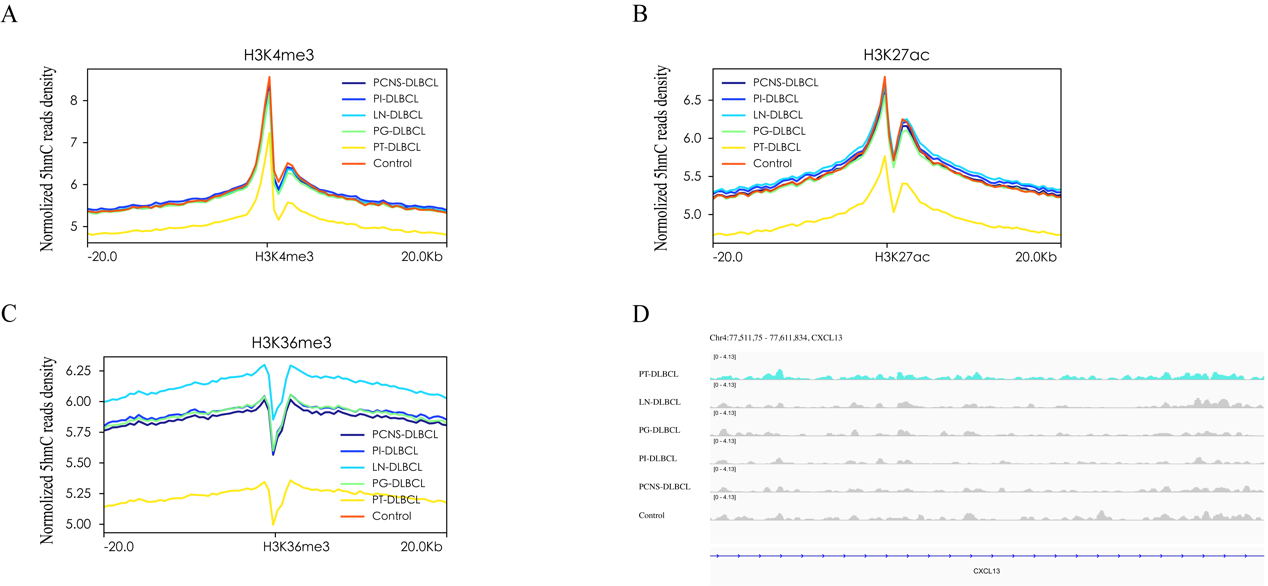
 Figure S2. Profiles of H3K4me3 (A), H3K27ac (B) and H3K36me3 (C) modifications around distal hMRs in the six groups samples. D. IGV visualization of the 5hmC signals of CXCL13 gene


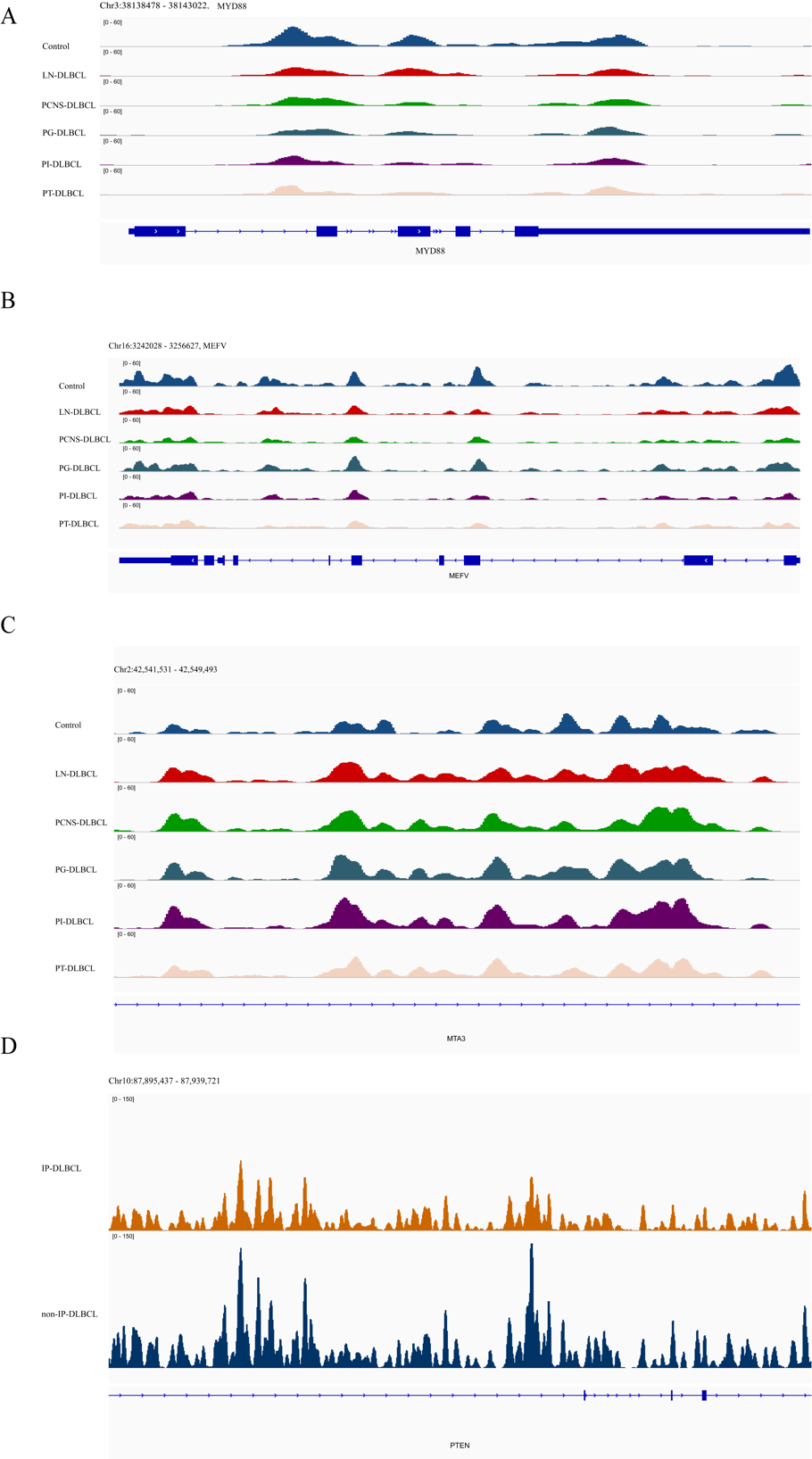


Figure S3. IGV visualization of the 5hmC signals of MYD88 gene (A) and MEFV gene（B） and surrounding regions in six groups. (C) IGV visualization of the 5hmC signals nearby MTA3 on chromosome 2 in the six groups. (D) IGV visualization of the 5hmC signals nearby PTEN gene on chromosome 10 in IP-DLBCL group and non-IP-DLBCL group.


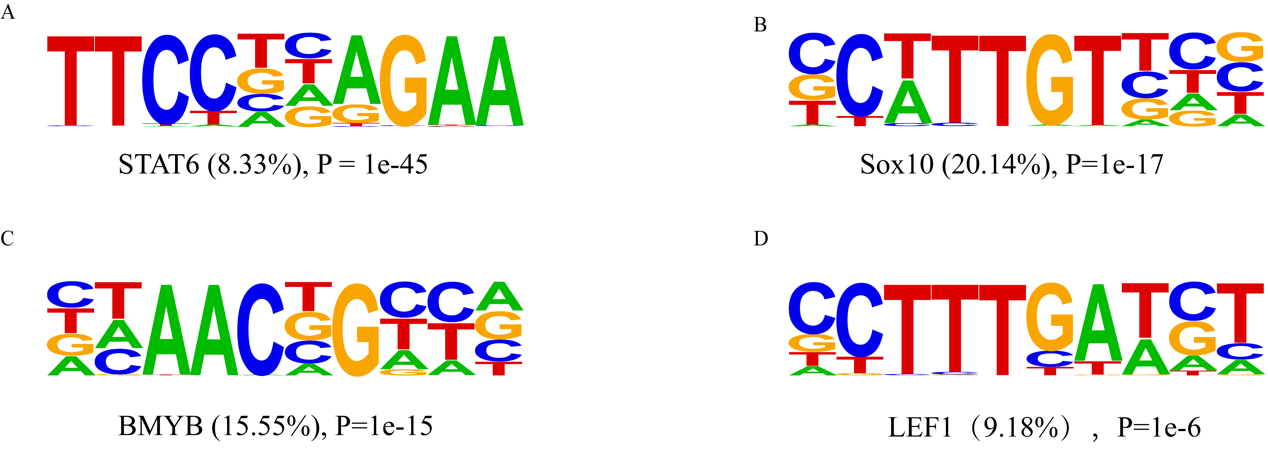


Figure S4. Top enriched known transcription factor binding motifs detected in psDhMRs in IP-DLBCL and non-IP-DLBCL groups. Motif information was obtained from the Homer motif database. The value in parenthesis represents the percentage of target sequences enriched with the binding motif of the indicated transcription factor.

Table S1. Demographic and baseline clinical characteristics of patients with N-DLBCL and EN-DLBCL.

| Characteristics | n (%) | | | P |
| --- | --- | --- | --- | --- |
|  | All patients | N-DLBCL | EN-DLBCL |  |
| Total | 216 | 47 | 169 |  |
| Gender |  |  |  | 0.032 |
| Female | 77 | 23 | 54 |  |
| Male | 139 | 24 | 115 |  |
| Age |  |  |  | 0.150 |
| ≤60 | 95 | 25 | 70 |  |
| >60 | 121 | 22 | 99 |  |
| B symptoms |  |  |  | 0.144 |
| Absence | 127 | 32 | 95 |  |
| Presence | 89 | 15 | 74 |  |
| ECOG-PS |  |  |  | 0.002 |
| 0 or 1 | 104 | 32 | 72 |  |
| ≥2 | 112 | 15 | 97 |  |
| Bulky disease |  |  |  | 0.286 |
| Absence | 177 | 41 | 136 |  |
| Presence | 39 | 6 | 33 |  |
| cell of origin |  |  |  | 0.672 |
| GCB | 64 | 16 | 48 |  |
| Non-GCB | 139 | 31 | 108 |  |
| Missing | 13 | 0 | 13 |  |
| Ki-67 index (%) |  |  |  | 0.153 |
| < 70 | 27 | 9 | 18 |  |
| ≥70 | 181 | 38 | 143 |  |
| Unknown | 8 | 0 | 8 |  |
| LDH |  |  |  | 0.297 |
| Normal | 148 | 36 | 112 |  |
| Elevated | 62 | 11 | 51 |  |
| Missing | 6 | 0 | 6 |  |
| IPI score |  |  |  | 0.624 |
| ≤2 | 181 | 41 | 140 |  |
| >2 | 32 | 6 | 26 |  |
| Missing | 3 | 0 | 3 |  |

Abbreviations: ECOG-PS: Eastern Cooperative Oncology Group (ECOG) performance status (PS); GCB: germinal center B-cell-like; LDH: lactate dehydrogenase; IPI: international prognostic index.

Table S2. Clinical characteristics of DLBCL patients and healthy controls

| Cases | Primary site | gender/age | cell of origin | Ki-67(%) | LDH | IPI |
| --- | --- | --- | --- | --- | --- | --- |
| LN-1 | Lymph nodes | Male/63 | GCB | 80 | Elevated | 3 |
| LN-2 | Lymph nodes | Male/56 | nonGCB | 50 | Normal | 2 |
| LN-3 | Lymph nodes | Female/64 | GCB | 60 | Normal | 1 |
| LN-4 | Lymph nodes | Female/51 | nonGCB | 60 | Elevated | 1 |
| LN-5 | Lymph nodes | Male/48 | nonGCB | 70 | Normal | 1 |
| PT-1 | Testis | Male/39 | nonGCB | 90 | Elevated | 1 |
| PT-2 | Testis | Male/80 | nonGCB | 90 | Elevated | 3 |
| PT-3 | Testis | Male/76 | nonGCB | 60 | Normal | 1 |
| PT-4 | Testis | Male/54 | nonGCB | 90 | Elevated | 1 |
| PT-5 | Testis | Male/65 | nonGCB | 90 | Normal | 1 |
| PCNS-1 | Central nervous system | Male/61 | GCB | 60 | Normal | 2 |
| PCNS-2 | Central nervous system | Male/28 | GCB | 90 | Elevated | 2 |
| PCNS-3 | Central nervous system | Female/58 | nonGCB | 90 | Normal | 1 |
| PI-1 | Intestine | Male/72 | GCB | 90 | Elevated | 3 |
| PI-2 | Intestine | Male/52 | GCB | 50 | Normal | 2 |
| PI-3 | Intestine | Female/63 | nonGCB | 80 | Normal | 2 |
| PG-1 | Gastric | Female/52 | nonGCB | 95 | Normal | 1 |
| PG-2 | Gastric | Female/60 | GCB | 80 | Normal | 0 |
| PG-3 | Gastric | Female/47 | GCB | 60 | Normal | 0 |
| PG-4 | Gastric | Female/77 | nonGCB | 80 | Normal | 2 |
| Ctrl-1 | Healthy individual | Male/55 | NA | NA | NA | NA |
| Ctrl-2 | Healthy individual | Female/47 | NA | NA | NA | NA |
| Ctrl-3 | Healthy individual | Male/50 | NA | NA | NA | NA |
| Ctrl-4 | Healthy individual | Female/42 | NA | NA | NA | NA |

Table S3. Sequencing technical details including sequencing depth, mapping rate, and PCR duplication rates in 24 cases.

|  | rawdata | mappable reads | mapping ratio | unique reads | unique ratio | mean depth |
| --- | --- | --- | --- | --- | --- | --- |
| health control-1 | 42,172,748 | 21,755,423 | 51.59% | 8,970,007 | 41.23% | 2.84248 |
| health control-2 | 44,887,385 | 22,631,834 | 50.42% | 12,598,724 | 55.67% | 2.96262 |
| health control-3 | 41,517,181 | 24,203,035 | 58.30% | 16,916,818 | 69.90% | 3.28381 |
| health control-4 | 40,941,548 | 37,719,319 | 92.13% | 15,801,445 | 41.89% | 3.8066 |
| LN-DLBCL-1 | 33,902,260 | 31,440,409 | 92.74% | 20,489,949 | 65.17% | 4.51659 |
| LN-DLBCL-2 | 36,384,792 | 33,616,996 | 92.39% | 20,679,167 | 61.51% | 4.67426 |
| LN-DLBCL-3 | 32,812,465 | 30,682,615 | 93.51% | 21,983,589 | 71.65% | 4.86305 |
| LN-DLBCL-4 | 31,352,799 | 28,238,504 | 90.07% | 14,703,591 | 52.07% | 3.57388 |
| LN-DLBCL-5 | 32,555,426 | 29,745,037 | 91.37% | 18,878,888 | 63.47% | 3.8994 |
| PCNS-DLBCL-1 | 30,698,636 | 27,873,280 | 90.80% | 14,207,025 | 50.97% | 3.60731 |
| PCNS-DLBCL-2 | 40,213,004 | 37,041,231 | 92.11% | 16,914,450 | 45.66% | 3.8635 |
| PCNS-DLBCL-3 | 39,674,003 | 36,323,369 | 91.55% | 19,045,060 | 52.43% | 4.28881 |
| PG-DLBCL-1 | 36,155,740 | 20,248,942 | 56.00% | 12,174,608 | 60.12% | 2.75959 |
| PG-DLBCL-2 | 32,115,252 | 29,696,788 | 92.47% | 11,546,764 | 38.88% | 3.31059 |
| PG-DLBCL-3 | 46,673,413 | 43,412,218 | 93.01% | 25,211,795 | 58.08% | 5.16652 |
| PG-DLBCL-4 | 37,180,710 | 17,968,250 | 48.33% | 7,658,331 | 42.62% | 2.64002 |
| PI-DLBCL-1 | 41,376,478 | 38,093,509 | 92.07% | 22,660,063 | 59.49% | 5.16643 |
| PI-DLBCL-2 | 42,228,893 | 38,658,745 | 91.55% | 13,921,685 | 36.01% | 3.49954 |
| PI-DLBCL-3 | 35,400,463 | 32,410,697 | 91.55% | 7,440,273 | 22.96% | 2.81982 |
| PT-DLBCL-1 | 45,938,286 | 20,534,471 | 44.70% | 12,863,126 | 62.64% | 2.66535 |
| PT-DLBCL-2 | 44,941,671 | 41,415,333 | 92.15% | 20,575,877 | 49.68% | 4.00112 |
| PT-DLBCL-3 | 35,251,740 | 31,811,895 | 90.24% | 12,528,850 | 39.38% | 2.97511 |
| PT-DLBCL-4 | 34,754,475 | 31,795,628 | 91.49% | 12,630,562 | 39.72% | 3.08679 |
| PT-DLBCL-5 | 55,296,236 | 32,026,551 | 57.92% | 22,188,301 | 69.28% | 3.71892 |

Table S4. The details of the probe sequences.

| ID NO. | Sequence (5'-3') | Annotation |
| --- | --- | --- |
| 1 | CTACGCAAACTGGCTGTCAAAGTAACTG  ACCAGATCTCTCGGCTCTCTTGAGGCTA  CTGAGTTATCATGGACGCTA CCTCACAG | Ref spike-in, dsDNA |
| 2 | CTACGCAAACTGGCTGTCAA | qPCR-Ref-F |
| 3 | CTGTGAGGTAGCGTCCATGA | qPCR-Ref-R |
| 4 | TATAACCCGACGACTCGACCAGTAACTG  ACCAGATCTCTCGTGCCTCTTGAGGCTA  CTGAGTTAAGTGCAACATTGGGGCTAAC  CATCGATAGCATCCG5hmCCACAGGCAGT  GAGGCTACTGAGTCAGGCCATTGATGCAT  CTTTCCGAC | 5hmC spike-in,dsDNA |
| 5 | TATAACCCGACGACTCGACC | qPCR-5hmC-F |
| 6 | GTTAGCCCCAATGTTGCACT | qPCR-5hmC-R |
